# 干果类食品的样品前处理与分析检测方法研究进展

**DOI:** 10.3724/SP.J.1123.2021.06030

**Published:** 2021-09-08

**Authors:** Lihui ZHOU, Xiaohua XIAO, Gongke LI

**Affiliations:** 中山大学化学学院, 广东 广州 510275; School of Chemistry, Sun Yat-sen University, Guangzhou 510275, China; 中山大学化学学院, 广东 广州 510275; School of Chemistry, Sun Yat-sen University, Guangzhou 510275, China; 中山大学化学学院, 广东 广州 510275; School of Chemistry, Sun Yat-sen University, Guangzhou 510275, China

**Keywords:** 样品前处理, 分析检测方法, 食品安全, 有害物质, 干果, 综述, sample preparation, analytical methods, food safety, hazardous substances, dried fruit foods, review

## Abstract

坚果、果脯等干果类食品含有丰富的营养成分,深受国内外广大消费者的喜爱。但这些食品在果实生产、加工、储运时会使用农药或产生霉变等,造成干果中农药、重金属、霉菌毒素或添加剂等有害成分残留,甚至超过国家限量要求,带来严重的食品安全问题。因此,加强干果类食品的质量监督具有重要的经济和社会意义。但干果类食品基质复杂,有害物质种类多,结构和性质差异大,含量低,其分析检测需要快速高效的样品前处理技术和准确灵敏的分析检测方法。该文主要综述了近十年来干果类食品中有害物质的样品前处理及分析检测方法研究进展。其中样品前处理方法主要包括各种场辅助萃取法、相分离法和衍生化萃取方法等。场辅助萃取法主要是借助超声波和微波场等外场(协同)作用加快干果中有害物质的溶出速度,提高其萃取效率。相分离法,包括固相(微)萃取、分散固相萃取和液相(微)萃取法等,具有溶剂消耗少、分离富集效率高的优势,是干果样品分析中较常使用的前处理方法。该文还重点介绍了干果中各类有害成分分析检测技术,主要包括色谱、原子光谱、无机质谱、电化学分析等常规实验室方法,以及一些适用于现场分析的快速检测技术,并以此为基础,展望了干果类食品中有害物质分析检测技术的发展趋势。

## Abstract (en)

Dried fruit foods, including nuts and preserved fruits, are favored by consumers and are rich in protein, lipids, minerals, vitamins, and other nutrients. However, these food products can be contaminated by pesticide residues, heavy metals, mycotoxins, and additives during growth, processing, storage, and transportation. The presence of such pollutants in excess of a certain limit will lead to food safety problems. Therefore, it is of great economic and social significance to strengthen the quality supervision of dried fruit foods. However, these foods have a complex matrix and low concentrations of various harmful substances, which necessitates efficient and appropriate sample preparation methods as well as rapid, accurate detection methods. In the present article, the sample preparation and analytical methods for harmful substances in dried fruit foods since 2010 are reviewed. The sample preparation methods are classified as field-assisted extraction, phase separation, and derivatization and extraction methods. The field-assisted extraction method is based on the action of an external field (synergistic) such as ultrasonic or microwave fields to increase the dissolution rate of hazardous substances in dried fruits and improve the extraction efficiency. Phase separation methods such as solid-phase extraction, dispersive solid-phase extraction and solid-phase microextraction are commonly used as sample preparation methods for dried fruit samples, because of the advantages of low solvent consumption and wide analysis range. Moreover, this paper discusses the progress of various analytical methods for these hazardous substances in dried fruits, including conventional laboratory methods such as chromatography, atomic spectroscopy, inorganic mass spectrometry, and electrochemical analysis, as well as rapid detection techniques suitable for field analysis. Laboratory testing has the advantages of high accuracy, high sensitivity, and low detection limits. However, it has the disadvantages of complicated preparation, long analysis time, and difficult operation. Rapid detection technology speeds up the analytical speed, has operational simplicity, and saves analysis time. The complexity of the food matrix, which easily interferes with the sample matrix, low selectivity, and difficulty in accurate quantification, it is necessary to minimize cases of incorrect or erroneous detection. Therefore, rapid detection of harmful substances in dried fruit foods is possible by optimizing the sample pretreatment methods and detection technologies, and by seeking new (especially, on-site) detection technologies. Prospects on the development of selective and non-destructive sample preparation methods and automated, high-throughput, rapid detection methods in dried fruit food analysis are presented. The development of new, green rapid sample pretreatment methods and technical products that integrate separation, enrichment, and detection as well as the construction of accurate and sensitive rapid detection methods are expected to become the development trend in the analysis of harmful substances in dried fruit foods.

干果类食品,包括坚果、果干和果脯等,来源广泛,种类多样,具有良好的口感和营养价值,易于储存和携带^[1]^,深受国内外消费者的喜爱。但干果在果实生产、食品加工与储存运输等各个环节容易产生或带入各种有害物质,包括生产环境中农药和重金属等有害物质的使用和迁移累积^[2,3]^,食品加工过程中各种添加剂的过量或违规使用^[4]^,储存过程中因霉变等因素带来的霉菌毒素滋长^[5,6]^以及产品自身的致敏源等。消费者食用含有各种有害物质的干果食品容易出现安全问题,如农药残留会干扰内分泌,引起中毒,甚至致癌、致突和致畸^[7,8]^;重金属元素可通过降水、尘埃沉降和人类活动等方式进入土壤,并通过食物链迁移到人体,对人类健康构成直接或间接的威胁^[9,10,11,12]^。葡萄干比其他干果特别是带壳的干果更容易被霉菌毒素污染^[13,14]^,此外,多种霉菌毒素还可能产生协同/累加作用,损伤肝脏和免疫系统^[15]^;食品添加剂主要包括各类防腐剂、甜味剂、着色剂等,超量使用会引发致畸、致癌、中毒等风险^[16,17]^。过敏性疾病已成为全球第六大疾病,致敏源可导致食物过敏^[18]^。

干果类食品基质复杂,有害物质种类多,结构和性质差异大,含量低,其分析检测需要有快速高效的样品前处理技术和准确灵敏的分析检测方法。干果果肉的低含水率会阻碍萃取溶剂渗透,其较高含糖量也容易产生包覆现象,使农残等分析物溶解不完全而萃取效率低;色素、有机酸等杂质的干扰也会影响样品分析的准确度和灵敏度。近年来干果类食品中常用的样品前处理方法有微波辅助萃取法(MAE)^[19]^、超声波辅助萃取法(UAE)^[20]^等场辅助方法,固相(微)萃取法(SP(M)E)^[21,22,23]^、分散固相萃取法(DSPE)^[24]^、(分散)液液微萃取法(DLLME)等相分离法,以及衍生化方法等;干果类食品有害物质分析以色谱法^[25,26,27]^等实验室检测方法为主,一些快检技术^[28,29]^也引起了广泛研究。表1总结了干果中常见有害物质的种类、名称、限量标准和分析检测技术。本文综述了干果类食品有害物质的样品前处理方法和分析检测研究进展。

**表1:** 干果类食品中有害物质的种类、名称、限量标准和分析检测方法

Hazardous substances	Category	Analytes	Maximum residue limit/ (mg/kg)		Maximum usage/ (g/kg)		Sample preparation methods	Analytical methods	Ref.
Pesticide	insecticide	pyrethroids, organic phosphines	0.01-	6	-		UAE, MAE,	GC, GC-MS,	[30-34]
residue	bactericide	dithiocarbamates, triazoles	0.01-	60	-		SPE, DSPE,	HPLC-MS/MS,	
	herbicide	paraquat, aquacide, glufosinate ammonium	0.01-	0.3	-		QuEChERS	HPLC	
	plant growth regulator	gibberellin, forchlorfenuron, bentazone, zearalenone, 2,4-dichlorophenoxyacetic acid	0.2-	10	-				
Heavy metal	heavy metal	Pb, Hg, Cd, Cr, As	-		-		Microwave- digestion, UAE	AAS, AFS, ICP- OES, ICP-MS	[35]
Fungimycin	fungimycin	aflatoxin	5.0-	50^*^	-		SPE, SPME, DSPE, QuEChERS, LLME	HPLC, HPLC-MS, ELISA, FL, ECL	[36-38]
Food additives	sweetening agent	aspartame, saccharin sodium, cyclamate	-		0.025-	6.0	UAE, SPE, derivatization,	IC, HPLC, HPLC- MS, UV-Vis	[39,40]
	preservative	nitrite	-		0.5-	1.0	DLLME	GC, HPLC, UV-Vis,	[16,39,41]
	decolorant	sulfur dioxide, sulfur	-		0.05-	0.35		ELISA, SERS, IC, SWSV	
	colorant	sudan red, rose red B, acid orange Ⅱ	-		0.1-	10		CE, ICP-MS, HPLC, SERS	[39,42,43]
Allergen	allergenic protein	Cora 1, Cora 8, Arah 1	-		-		UAE	ELISA, PCR, HPLC-MS/MS	[44,45]

UAE: ultrasound assisted extraction; MAE: microwave assisted extraction; DSPE: dispersive solid phase extraction; LLME: liquid-liquid microextraction; DLLME: dispersive liquid-liquid microextraction; AAS: atomic absorption spectroscopy; AFS: atomic fluorescence spectrometry; ICP-OES: inductively coupled plasma-optical emission spectrometry; ICP-MS: inductively coupled plasma mass spectrometry; ELISA: enzyme linked immunosorbent assay; FL: fluorescence; ECL: electrochemiluminescence; IC: ion chromatography; UV-Vis: ultraviolet and visible spectrophotometry; SERS: surface-enhanced Raman scattering; SWSV: square wave stripping voltammeter; CE: capillary electrophoresis; PCR: polymerase chain reaction; -: no data. * The unit is μg/kg.

## 1 干果类食品样品前处理方法

干果类食品前处理方法包括场辅助前处理方法、相分离前处理方法及衍生化前处理方法。表2概括了干果食品分析中的不同样品前处理方法的优缺点。

**表2:** 干果类食品样品前处理方法及其优缺点

Sample preparation methods	Advantages	Disadvantages	Ref.
UAE, MAE	high extraction efficiency, fast speed,	filtration, operation trouble	[46,47]
	low cost, low solvent consumption		
SPE	low solvent consumption, no separation operation, small volume samples	long extraction time, poor batch repeatability	[48]
SPME	combination of extraction and concentration, time-consuming, few organic solvent	fragile fiber, peeling of coating, memory effect	[49]
DSPE	simple and fast operation, low cost, wide analysis range	cumbersome process, matrix effects, low-throughput analysis	[50]
LLME DLLME	high extraction efficiency, environmentally friendly	centrifugal separation, poor recovery	[51] [52]
Derivatization	improvements in the detectability of analytes and separation effect	cumbersome operation, effects on chromatographic separation and quantitative accuracy	[53]

### 1.1 场辅助样品前处理法

场辅助萃取法主要包括超声波辅助萃取法(UAE)和微波辅助萃取法(MAE)等,在干果中添加剂和农药残留检测方面应用较多。UAE通常使用乙醇水溶液、氢氧化钠溶液作为溶剂,用于萃取果脯中的罗丹明B^[46]^和亚硫酸盐^[54]^,可有效降低果胶和多糖干扰,提高检测灵敏度。UAE操作简单方便,常用于样品中微量分析物的快速萃取,也可以通过循环提取或连续提取等方式处理大量的样品。MAE通过微波作用强化传热和传质效率,提高萃取性能,与传统浸渍提取和索式抽提法相比,具有快速高效、环境友好等特点^[55]^。MAE已应用于干果中多酚类化合物等有效成分的萃取分离^[47]^,萃取效率受萃取剂、萃取温度和萃取时间的影响较大。经优化后的MAE与其他萃取方法相比,其萃取效率可提高8.4%~34.8%,且能耗更低。

超声波、微波等场作用可与各种相分离方法,如SPE、SPME等结合,进一步提高或者改善其萃取分离效率。陈正毅等^[56]^以水为溶剂超声波辅助萃取干果中的糖精钠后,SPE对其进行净化富集,结合表面增强拉曼光谱(SERS)分析,方法检出限为0.6 g/kg,灵敏度高,杂质干扰小。

### 1.2 相分离样品前处理法

1.2.1 固相萃取法

SPE常用来提取瓜子、果脯蜜饯、葡萄干等食品中的添加剂^[48]^和农残^[57]^,也是目前食品中霉菌毒素^[58]^的主要前处理方法。SPE主要以C_18_为填料,石油醚、丙酮为萃取溶剂,常用洗脱液是乙腈、甲醇和乙醇与水的混合溶液。

SPE可同时完成样品富集和净化,提高检测灵敏度,它与液相色谱在线联用可实现自动化并简化分离步骤,减少样品损耗,特别适用于常规分析大量样品,在食品、环境等领域应用越来越广。Campone等^[59]^将加压液体萃取与SPE结合,开发出一种在线样品前处理装置,用于分析干果中的黄曲霉毒素。该装置可同时进行富集和净化,与色谱等分析技术联用后,整个分析过程可实现完全自动化,简化了样品处理步骤,实现了高通量分析,并取得了高度精确的结果。

1.2.2 固相微萃取法

SPME集萃取、浓缩于一体,可大大加快样品分离分析的效率。但传统SPME存在萃取纤维易断裂、涂层剥离和记忆效应等缺点,为此开发出了一些新材料用于固相微萃取。Es’haghi等^[60]^通过溶胶-凝胶法制备碳纳米管溶胶纤维,使用中空纤维固相微萃取技术(HF-SPME)萃取、预富集花生样品中黄曲霉毒素B1(AFB1)和黄曲霉毒素B2(AFB2),具有很高的富集因子和良好的选择性。

管内SPME采用内表面涂层的开口管状熔融石英毛细管作为SPME器件,易与LC-MS在线耦合,萃取过程自动化,减少了分析时间,比手动/离线技术提供了更好的精密度、准确度和灵敏度。采用自动管内固相微萃取法萃取干果中真菌毒素时,样品无需处理,可直接将GC毛细管柱用作管内SPME装置,并将其放置在自动进样器的进样环和进样针之间,从而实现在线分离^[61]^,可成为食品中真菌毒素监测的有力工具。

顶空固相微萃取法(HS-SPME)适合挥发性成分如呋喃类物质的分离富集,可有效减少基体中干扰物质的影响^[49]^。萃取前向样品溶液中加入NaCl可降低样品中有机分析物的溶解度,增加样品基体与涂层吸附剂之间的吸附作用,提高萃取效率。

1.2.3 分散固相萃取法

DSPE操作简单快速,成本低,分析范围广,但容易受基体效应干扰,难以达到高通量分析的要求。当以聚酰胺为吸附剂,乙醇-氨溶液(8:2,v/v)为洗脱溶剂时,可有效去除无机盐和有机化合物对样品中着色剂的分析干扰^[62]^。由于传统吸附剂如丙基乙二胺(PSA)和C_18_选择性不强、效率有限,近年来一些碳材料,包括碳纳米管(CNTs)、活性炭纤维(ACFS)受到了关注。Singh等^[50]^使用镍纳米粒子和碳纳米纤维分散的活性炭纤维(Ni-ACF/CNF)作为吸附剂净化脂肪基质中的农药残留。结果表明,结晶碳和无定形碳的Ni-ACF/CNF组合可更好地清除脂肪酸基质,吸附效率比PSA和C_18_高。磁性纳米颗粒(MNPs)广泛用于食物、环境中微痕量污染物的磁分离富集。Karami-Osboo等^[63]^开发了一种分散磁性固相萃取技术,使用Fe_3_O_4_ MNPs去除杂质,使用微升级的氯仿萃取坚果中黄曲霉毒素,萃取效率高,操作方便。

QuEChERS法是农药残留和真菌毒素分析中常用的样品前处理方法。采用QuEChERS法萃取坚果中的农药残留时,使用乙腈作为萃取溶剂可有效去除脂肪、蛋白质等杂质的影响,萃取效率高^[64]^。但糖在乙腈中具有很高的溶解度并存在基质干扰,此法不适合含糖量高的样品。对QuECHERS进行自动多塞过滤清除(multi-plug filtration cleanup, m-PFC)改进,可减少手动操作,精确控制推拉循环的体积和速度^[65]^。m-PFC将多壁碳纳米管(MWCNT)与其他吸附剂和无水硫酸镁混合在一起,填充并萃取,不需要额外的涡旋或离心步骤即可同时富集干果中的多种农药残留。以锆为基础的改性硅胶吸附剂Z-Sep可起路易斯酸的作用,与电子供体相互作用;C_18_通过烷基链的疏水作用与甘油三酯相互作用,可有效去除脂肪。将Z-Sep与C_18_混合使用时可有效消除干扰,高效富集干果食品中的16种真菌毒素^[66]^。此外,将QuEChERS法与其他样品前处理方法如DLLME结合使用,分析物回收率得到显著改善^[67]^。

1.2.4 (分散)液-液微萃取法

液-液萃取法(LLE)是经典的样品前处理方法,但需要消耗大量有机溶剂,LLME和DLLME是基于LLE原理发展起来的,用于处理痕量组分的前处理技术^[51]^。Karami-Osboo^[52]^将Fe_3_O_4_纳米粒子作为胶体悬浮剂发展了一种纳米流体空气辅助分散液-液微萃取方法,只使用少量有机溶剂和纳米颗粒,即可完成葡萄干样品中曲霉毒素A的高效分离富集。超分子溶剂等新型溶剂在微萃取中的应用越来越多,Caballero-Casero等^[68]^以癸酸/四丁基葵酸铵囊泡作为超分子溶剂,提出了一种无溶剂微萃取方法,用于分离干果果实中的赭曲霉毒素A,该方法不需要稀释及净化步骤,基体干扰少,效率高。

### 1.3 衍生化样品前处理法

衍生化法是一种采用衍生反应把分析物转化成结构类似物的化学转换样品前处理方法。化学衍生法是衍生化的一种重要方法,其借助化学反应将分析物接上某种特定基团,从而改善其分离效果和检测灵敏度。Yu等^[53]^将甜蜜素钠与次氯酸钠反应转化为*N*,*N*-二氯环己胺,利用其较强的电负性,使用气相色谱-电子捕获检测器进行测定。该方法样品制备简单,衍生产物稳定性高,选择性好,甜蜜素的定量不受基体效应的影响。Robbins等^[69]^使用甲醛溶液将亚硫酸盐转化为羟甲基磺酸盐(HMS),然后用C_18_ SPE柱去除亲脂性化合物,并使用亲水相互作用色谱柱将HMS与其他基质组分分离,建立了高效灵敏的LC-MS/MS方法。

## 2 干果类食品中有害物质的分析检测

食品中的有害物质检测方法一般包括实验室检测和快速检测两类,其中实验室检测通过使用色谱、原子吸收光谱(AAS)等一系列精密仪器来分析目标组分,具有准确、灵敏度高、检出限低、能够定性定量分析的优势,但存在前处理过程较复杂、分析时间长、仪器昂贵、操作难度大等不足。快速检测方法具有检测时间短、操作简单、价格便宜等优势,但其检出限较高、灵敏度较低,主要起到半定量检测和初筛作用。下文总结了近年来干果类食品中的分析检测方法。

### 2.1 实验室检测方法

2.1.1 色谱法

HPLC广泛用于干果中甜味剂^[70]^、防腐剂^[71]^以及呋喃化合物^[51]^等的分析检测,是食品中着色剂分析的标准方法^[72]^。Geng等^[73]^对HPLC的荧光检测器进行改进,设计了一种新型紫外发光二极管诱导荧光检测器(LED-IF),用于分析食品中的黄曲霉毒素。该检测器使用普通紫外LED作为激发光源,采用光电放大器代替光电倍增管进行荧光检测,实现了与传统Xe灯为激发光源的荧光检测相同的灵敏度,大大降低了成本。

GC常用于干果中有机氯、有机磷、拟除虫菊酯类、氨基甲酸酯类农药残留^[74]^以及甜味剂^[75]^的检测。Yu等^[53]^将甜蜜素衍生后用气相色谱-电子捕获检测器(GC-ECD)分析,果脯中防腐剂、工业染料等其他食品添加剂干扰小,方法检出限为0.25 mg/kg。

将样品前处理技术与LC-MS结合,可以减少样品处理时间和溶剂消耗,实现高通量分析。LC-MS常被用来定性、定量分析干果中的农药残留^[76]^、漂白防腐剂^[69]^、合成染料^[77]^、真菌毒素^[78]^和致敏源^[79,80]^。Alsharif等^[81]^建立了干果、坚果等120种食品中霉菌毒素的QuEChERS-LC-MS/MS方法。他们应用单变量-多变量组合的化学计量学方法优化分析方法,缩短了分析时间,提高了电离效率,方法的回收率为81.94%~101.67%。

离子色谱法是食品中亚硫酸盐等离子化合物测定的常用方法。Liao等^[82]^建立了干果中游离亚硫酸盐的离子色谱法,当使用氢氧化钠溶液(pH>13)萃取时, ${\mathrm{SO}}_{3}^{2-}$ 均为游离形式,且可以稳定存在,不需要使用氧化剂和稳定剂,样品制备简单,分析时间为18 min,适用于干果中高含量亚硫酸盐的测定。

2.1.2 原子光谱法

AAS和原子荧光光谱法(AFS)是现今在食品、环境中重金属检测应用最广泛的分析方法。尤其是AFS,灵敏度高,检出限比AAS低,基体效应小,线性范围宽,谱线简单且干扰小^[83]^,但仅能分析砷、硒、铅、锡、汞等元素。电感耦合等离子体发射光谱仪(ICP-OES)中心气化温度高可以使样品充分气化,准确度高,可以实现连续快速多元素测定^[84]^。

2.1.3 无机质谱法

电感耦合等离子体质谱法(ICP-MS)具有高灵敏性的特点^[85]^,对于低含量样品分析的准确度比AAS以及AFS高^[86]^,也是食品中重金属检测的标准方法^[87]^,但此法成本高且易受污染,限制了其普遍应用。李文祥等^[88]^采用ICP-MS测定了蜜饯腌制前后的重金属含量,并分析了蜜饯腌制过程中重金属的迁移变化。此外,ICP-MS还可用于定量分析干果类食品中着色剂TiO_2_[89],采用内标法来消除基体干扰,确保分析结果准确可靠。

2.1.4 电化学分析法

电化学分析在着色剂和工业染料的检测中得到了广泛应用。电化学测定罗丹明B一般是测定苯环C=N基团的氧化信号,因此电极是影响分析性能的关键步骤。方波溶出伏安法(SWSV)是一种快速、高灵敏度和高效能的电化学分析方法。Zhang等^[90]^用氧化硅柱状磷酸锆/全氟磺酸复合材料(SPZP/NAF)修饰的玻碳电极测定罗丹明B, SPZP具有层状结构和较大的比表面积,表现出高的电催化活性。该电化学传感器的线性响应范围为0.01~5.0 μmol/L,检出限低至4.3 nmol/L,稳定性好。Yi等^[62]^结合DSPE和场放大进样-毛细管电泳-电容耦合非接触电导检测,建立了果脯样品中合成着色剂的高灵敏度分析方法,5种常见食用色素的检出限为0.035~0.055 mg/kg。部分实验室检测方法如表3所示。

**表3:** 干果中有害物质实验室检测方法

Analytical method	Analytes	Samples	LOD/(μg/kg)	Recovery/%		Ref.
SPME-LC/MS	patulin	dried fruit	0.0235^*^	92.5-	94.5	[91]
QuEChERS-UPLC-MS/MS	pesticide residue	nut	0.01-10	51.0-	126.0	[92]
HPLC	food additives	preserved fruit	100-250	90.2-	106.3	[93]
QuEChERS-GC-MS/MS	multi-residue pesticide	dried fruits	-	70-	120	[94]
QuEChERS-LC-ESI-MS	acrylamide	dried fruits	2.0	61-	82	[95]
DSPE-CE	synthetic food colorants	preserved fruit	3.50-5.50	94.3-	102	[62]
IC	sulfites	dried fruits	143^*^	81-	105	[82]
AFS	mercury	nuts	0.08	100-	101	[83]
ICP-MS	heavy metal	dried strawberry	2.60-427.60	79-	104.9	[85]

* The unit is ng/mL.

### 2.2 快速检测方法

2.2.1 分子光谱法

常见SO_2_快速检测方法是盐酸副玫瑰苯胺分光光度法,但此法中四氯化汞有剧毒,会对人体和环境造成一定的伤害^[96]^,目前盐酸副玫瑰苯胺法已停止使用^[97]^,替换方法为碘滴定法。

荧光分析法(FL)检测灵敏度高,适用于干果中痕量真菌毒素的检测。上转化纳米颗粒(UCNPs)具有独特的反斯托克斯发射特性,可以有效避免生物样品的背景干扰,消除假阳性信号,是制备荧光探针的优良材料,也是生物样品成像的理想发光纳米材料。Wang等^[98]^设计了一种通过适体修饰上转换纳米颗粒(UCNPs-aptamer)与金纳米颗粒构建的基于发光共振能量转移的适体传感器。该适体传感器由UCNP-aptamer充当发光供体,GNPs充当能量受体,可以避免其他黄曲霉毒素信号的干扰,具有很强的特异性。花生样品中AFB1的检出限为0.17 ng/mL,与传统的荧光测定法相比,该方法具有准确性高、灵敏度高、样品消耗低的优点。

SERS在真菌毒素^[99]^、甜味剂^[100]^和致敏源^[101]^检测中也得到了越来越多的应用。Gezer等^[102]^基于可生物降解且能被金纳米包覆的玉米蛋白设计了一种SERS传感器,并建立了花生中Ara h1蛋白的检查方法。通过Ara h1单克隆抗体对传感器表面进行功能化,首次在可生物降解的金/锌膜SERS平台上检测花生过敏原蛋白Ara h1。

电化学发光(ECL)结合了化学发光与电化学分析的优势,响应速度快、灵敏度高并克服了化学发光分析法中发光试剂不易保存,重现性差的缺点。Yan等^[103]^基于酶驱动的可编程3D DNA纳米花(EPDNs),设计了一种增强且稳定的电化学发光生物传感器,如图1所示,用于AFB1的超灵敏检测。利用EPDNs积累大量带正电荷的Ru(Ⅱ)络合物(Ru(bpy ${)}_{3}^{2+}$ ),提高方法灵敏度。即使存在痕量的AFB1,也产生清晰的视觉电化学发光信号。与传统的适体直接竞争方法相比,协同效应确保了适体被更有效、更充分地竞争和检测。

**图1:**
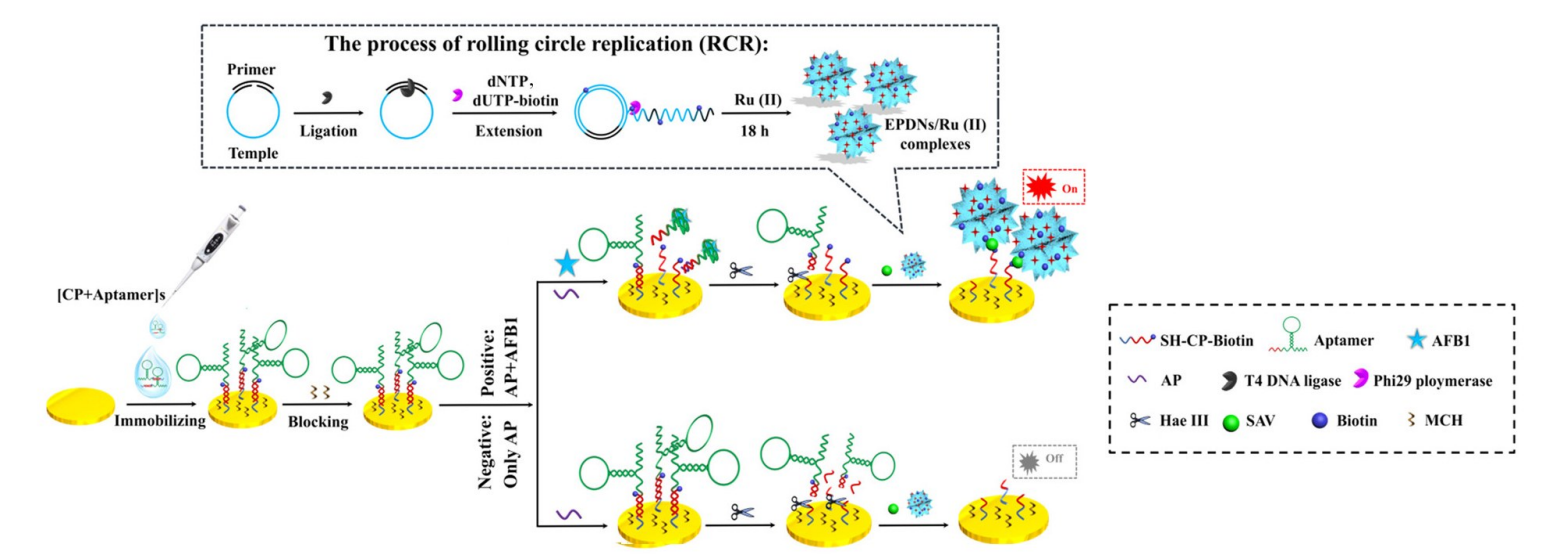
基于协同效应和酶促可编程3D DNA纳米花的电化学发光生物传感器原理图^[103]^

2.2.2 生物免疫法

生物免疫法主要基于免疫识别、抗体标记以及核酸杂交等技术发展起来的具有特异性的快检分析技术。在干果类食品中广泛应用于真菌毒素^[104]^和致敏源^[105,106]^的检测。

酶联免疫测定法(ELISA)包括夹心型和竞争型免疫分析两种形式,其中夹心型免疫测定法具有很高的灵敏度和特异性,但不适用于定量监测小分子,在干果中致敏源检测方面应用较多^[107]^。竞争型免疫测定法可以为小分子测定提供有利的形式,因为目标分析物可以通过其与标记半抗原/抗原竞争的能力进行监测。在传统的竞争型ELISA中,介导信号输出的酶通常作为信号探针使用^[108]^。实时聚合酶链式反应法(real-time PCR)是基于DNA靶标的检测方法,在热处理过程中仍具有稳定性,与商业ELISA测试相比,实时PCR特异性和灵敏性更高^[109]^,但DNA的存在不能保证蛋白质的存在,可能会出现假阳性结果。

光电化学传感是基于光电转化特性发展起来的新型检测技术,通过目标物与光电化学活性物质之间的相互作用或生物识别过程前后所引起的光电流(压)的变化与待测物浓度之间的关系进行定量分析。因为传统酶联免疫分析中使用的天然酶具有价格相对昂贵、易受到外界因素影响而失活等缺点。Lin等^[110]^设计了一种无需生物酶参与的新型光电化学免疫传感平台,用于定量检测食品中的AFB1。该工作利用银纳米粒子-标记AFB1-牛血清白蛋白(BSA)偶联物作为标记探针,与目标分析物AFB1竞争结合酶标板表面的相应抗体。利用硝酸溶解标记探针,释放出大量Ag^+^,引发其与固定在电极表面的CdTe量子点之间的离子交换反应,导致表面激子俘获的形成。形成的激子俘获可以降低修饰电极的光电流,基于此实现AFB1的定量分析。为了进一步克服光电化学免疫分析中常用的半导体(如CdTe)毒性高、稳定性差等缺点,Lin课题组^[111]^还提出了一种可以同时目视和光电化学分析的免疫传感平台,用于食品中AFB1的快速、灵敏检测。该工作同样采用了竞争型免疫分析平台,使得目标AFB1与偶联物葡萄糖氧化酶(GOx)-标记AFB1-BSA竞争结合修饰在磁珠表面的AFB1抗体,形成免疫复合物。免疫复合物中的GOx可以催化葡萄糖氧化,生成的H_2_O_2_进一步将电极表面的MnO_2_纳米片还原/刻蚀为Mn^2+^,导致修饰在电极表面的碳量子点(CQDs)解离。因此,MnO_2_-CQDs修饰电极的光电流信号会随着H_2_O_2_浓度增加而降低,所建立的光电化学免疫分析方法可检测低至2.1 pg/mL的AFB1。此外,根据MnO_2_纳米片解离前后MnO_2_-CQDs涂覆电极的颜色变化,还可以对AFB1进行目视检测。该工作建立的免疫分析方法具有良好的重现性和准确性,能够扩展到其他小分子或者真菌毒素的检测上,同时结合高通量微流控芯片装置,可以作为一个多功能免疫传感平台。

将微流控芯片技术应用于免疫分析方法中,可以显著减少样品成本及试剂用量,同时分析时间短、芯片尺寸小,是现场分析的理想选择。Angelopoulou等^[112]^首次设计了基于可容纳10个宽带马赫-森德干涉仪(BB-MZI)的硅微型传感器芯片,如图2所示,用于同时且无标记地测定4种过敏源,总检测时间仅为6.5 min,所获得的分析结果与ELISA的结果高度吻合。

**图2:**
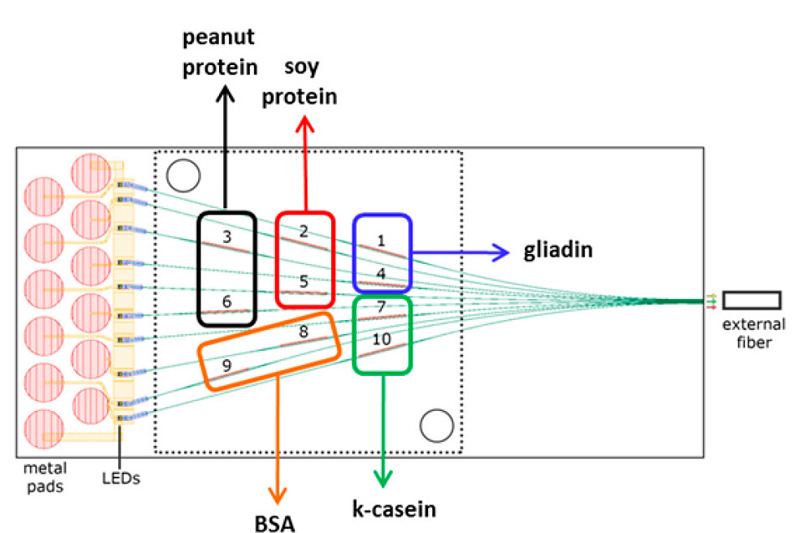
具有不同生物分子的BB-MZI对的芯片示意图^[112]^

## 3 总结与展望

近年来,干果类食品深受大众喜爱而食品安全风险较大,建立此类食品中有害物质的检测方法具有十分重要的意义。针对干果类食品的样品前处理方法,仍存在操作较为繁琐复杂、设备自动化智能化程度低等问题,开发选择性高、绿色无污染、成本低的前处理技术依然是干果类食品分析的重要研究内容,可通过发展新型微萃取方法、研制新型样品前处理装置或采用前处理-分析检测一体化技术来提高样品制备效率,缩短制备时间。微流控芯片技术因可在一块芯片上完成分离、富集、分析等多个步骤,在免疫分析、生物传感器等方面得到了越来越多的应用,开发出了许多超强运行能力的多功能芯片。干果中有害物质更多利用实验室方法进行检测,快速检测方法应用较少。因此可以通过优化样品前处理方法和检测技术,寻求新的检测方法,特别是现场检测技术来实现对干果类食品中有害物质的快速检测。但快检技术由于食品基体极其复杂、易受样品基体干扰、选择性不高、难以准确定量,导致出现“检不出、检不准、检不快”等瓶颈问题,在实际操作中需克服准确性与省时性、灵敏度和特异性、漏检率与错检率3方面的矛盾。研发集分离、富集、检测于一身的高灵敏、高通量与智能化的绿色快速样品前处理新方法与技术产品,构建精准、灵敏的快速检测方法,研制配套试剂与设备,有望成为干果类食品中有害物质分析的发展趋势。
